# CRM646-A, a Fungal Metabolite, Induces Nucleus Condensation by Increasing Ca^2+^ Levels in Rat 3Y1 Fibroblast Cells

**DOI:** 10.4014/jmb.1908.08043

**Published:** 2019-11-11

**Authors:** Yukihiro Asami, Sun-Ok Kim, Jun-Pil Jang, Sung-Kyun Ko, Bo Yeon Kim, Hiroyuki Osada, Jae-Hyuk Jang, Jong Seog Ahn

**Affiliations:** 1 Anticancer Agent Research Center, Korea Research Institute of Bioscience and Biotechnology, Cheongju 286, Republic of Korea; 2 Natural Medicine Research Center, Korea Research Institute of Bioscience and Biotechnology, Cheongju 8116, Republic of Korea; 3 Department of Biomolecular Science, KRIBB School of Bioscience, University of Science and Technology, Daejeon 411, Republic of Korea; 4 Chemical Biology Research Group, RIKEN CSRS, Saitama 351-0198, Japan; 5 Kitasato Institute for Life Sciences, Kitasato University, Tokyo 108-8641, Japan

**Keywords:** Nucleus condensation, plasma membrane, Ca^2+^ signaling, Ca^2+^ ionophore-like agent, ERK pathway

## Abstract

We previously identified a new heparinase inhibitor fungal metabolite, named CRM646-A, which showed inhibition of heparinase and telomerase activities in an in vitro enzyme assay and antimetastatic activity in a cell-based assay. In this study, we elucidated the mechanism by which CRM646-A rapidly induced nucleus condensation, plasma membrane disruption and morphological changes by increasing intracellular Ca^2+^ levels. Furthermore, PD98059, a mitogen-activated protein kinase (MEK) inhibitor, inhibited CRM646-A-induced nucleus condensation through ERK1/2 activation in rat 3Y1 fibroblast cells. We identified CRM646-A as a Ca^2+^ ionophore-like agent with a distinctly different chemical structure from that of previously reported Ca^2+^ ionophores. These results indicate that CRM646-A has the potential to be used as a new and effective antimetastatic drug.

## Introduction

Microbial secondary metabolites produced by micro-organisms are an important source of natural products, many of which have been characterized as helpful study tools as well as candidates for new drugs. It can however cost a lot of time and money to find new bioactive molecules using common screening techniques. We previously isolated CRM646-A and -B, which are secondary metabolites produced by the soil fungus *Acremonium* sp. MT70646, while screening for heparinase inhibitors [1]. CRM646-A and -B have sugar moieties that are phenol glucuronides consisting of a dimeric 2,4-dihydroxy-6-alkylbenzoic acid; these moieties belong to the orcinol *p*-depside family and an aliphatic chain motif. The total synthesis of CRM646-A and -B was first reported by Wang *et al*. [2].

However, the cellular and molecular mechanisms underlying their biological activities are still unknown. It is sometimes difficult to reveal a mode of action directly from an activity-guided investigation, and thus it is necessary to design detailed assay systems [3-5]. In an effort to determine the mechanisms of biological activity, we screened these active compounds by using a rat 3Y1 fibroblast cell morphological change assay and were able to identify unique tubulin and actin inhibitors as well as visualize phenotypes, including disrupted cell–cell interaction, cellular elongation, and cellular outgrowth [6]. In the course of this investigation, CRM646-A was identified as a compound that quickly induced nucleus condensation, plasma membrane disruption and morphological changes by increasing intracellular Ca^2+^ levels. Here, we describe these novel functions of CRM646-A as a Ca^2+^ ionophore-like agent in rat 3Y1 fibroblast cells. Nucleus condensation induced by CRM646-A was associated with ERK1/2 activation by increasing intracellular Ca^2+^ levels.

## Materials and Methods

### Reagents and Antibodies

CRM646-A was prepared as previously described [1]. Paclitaxel and PD98059 were purchased from Calbiochem. A23187 and gramicidin were purchased from Sigma-Aldrich and Thermo Fisher Scientific (USA), respectively. Hoechst 33342, propidium iodide, FM1-43FX, fluorescent Mg^2+^ indicator (mag-fura-2), fluorescent Na^+^ indicator (CoroNa Green), and fluorescent Ca^2+^ indicator (Fluo-4) were purchased from Thermo Fisher Scientific. Anti-ERK1/2 antibody and anti-phospho-ERK1/2 (T202/Y204) were purchased from Cell Signaling Technology. Methanol (MeOH), dimethyl sulfoxide (DMSO), 3.7% formaldehyde, Triton X-100, bovine serum albumin (BSA), trypan blue solution, and Tween-20 were purchased from Sigma-Aldrich.

### Cell Lines and Culture

Rat 3Y1 fibroblast cells were cultured in Dulbecco’s Modified Eagle’s Medium (DMEM) supplemented with 10% fetal bovine serum (FBS) in the presence of 30 µg/ml of penicillin and 42 µg/ml of streptomycin under a humidified atmosphere of 5% CO_2_ at 37°C.

### Nuclear Staining with Hoechst 33342 and Propidium Iodide

Rat 3Y1 cells (3 × 10^4^ cells per well) were seeded on a 48-well microplate and incubated 24 h. The cells were treated with the indicated concentrations (30, 10, and 3 µM) of CRM646-A or 10 µM paclitaxel for 15 min. After 15 min, Hoechst 33343 and propidium iodide staining were performed under a fluorescence microscope. Cells were stained with 100 ng/ml of Hoechst 33343 and propidium iodide for 5 min at 24°C. The fluorescence was detected using an ECLIPSE Ti-U inverted microscope (Nikon Corporation).

### Cell Viability Assay

The effect of CRM646-A on cell viability was analyzed by using trypan blue exclusion method. Cells were seeded in a 48-well plate at 3×10^4^ cells/well and incubated overnight. The cells were treated with the indicated concentrations (30, 10, and 3 µM) of CRM646-A or 10 µM paclitaxel for 15 min. Cells were trypsinized, stained with trypan blue, and counted using a hemocytometer. Cell survival was expressed as percentage of viable cells to total cells. Values represent means ± SD for quadruplicate samples, and the experiments were repeated three times.

### Cell Membrane Staining

For immunofluorescence observation, 3Y1 cells were seeded on a microplate (3 × 10^4^ cells/ml) in a 48-well plate, cultured for 18 h, and treated with CRM646-A for 15 min. The medium was aspirated, and the cells were fixed with 3.7% formaldehyde in PBS for 15 min and permeabilized for 5 min with PBS containing 0.2%Triton X-100. After being washed with 1% BSA PBS, they were blocked with 10% FBS for 20 min. After being washed with 0.1%BSA PBS, the wells were treated with Alexa Fluor 488-conjugated FM1-43FX in 0.1% BSA PBS, placed in a humidified atmosphere of 5% CO_2_ at 37°C, and incubated for 1 h. After being washed with 0.1% BSA PBS, the wells were overlaid with 100 ng/ml of Hoechst 33343, incubated for 5 min at 24°C, and washed with 0.1% BSA PBS. The fluorescence was detected using an ECLIPSE Ti-U inverted microscope (Nikon Corporation, Japan).

### Detection of Fluorescence Imaging in Live Cells

3Y1 cells were seeded on a plate at 3 × 10^4^ cell/ml in a 48-well plate, cultured for 18 h, and treated with CRM646-A and control compounds (A23187 or gramicidin) for 15 or 30 min at 37°C, 5%CO_2_. We added a fluorescent Ca^2+^ indicator (Fluo-4), fluorescent Na^+^ indicator (CoroNa Green), and fluorescent Mg^2+^ indicator (mag-fura-2) to the appropriate wells, incubated for 15 min at 37°C at 5% CO_2_ and washed with DMEM/10% FBS. The fluorescence was detected using an ECLIPSE Ti-U inverted microscope (Nikon Corporation).

### Western Blotting Analysis

Pre-confluent 3Y1 cells were pretreated with or without 25 µM PD98059 containing 10% FBS DMEM medium for 1 h at 37°C at 5% CO_2_ and were untreated or exposed to 30 µM CRM646-A for 0, 5, 10 15 30, or 60 min. In 3Y1 cell western blotting experiments, after CRM646-A stimulation, 3Y1 cells were lysed in 0.1 mL of lysis buffer [250 mM Tris-HCl (pH 6.8), 8% sodium dodecyl sulfate, 40% glycerol, and 20% 2-mercaptoethanol]. Lysis samples were boiled at 100°C for 5 min, and the resulting total cell lysates were western blotted. For western blotting, the cell lysates were subjected to SDS-PAGE and transferred onto polyvinylidene difluoride membrane (Millipore). After transfer, the blots were incubated with blocking solution (5% skim milk) in washing buffer [20 mM Tris-HCl (pH 7.4), 8% NaCl, and 0.0005% Tween-20] and probed with primary antibodies anti-phospho-ERK1/2 and anti-ERK1/2 diluted in the blocking solution. These signals were visualized using HRP-conjugated secondary antibodies and enhanced chemiluminescent substrate (PIERCE) according to the manufacturer’s instructions. The results shown are representative of three experiments.

## Results

### Fluorescence Imaging of Rat 3Y1 Fibroblast Cells after Exposure to CRM646-A

**Fig. 1:**
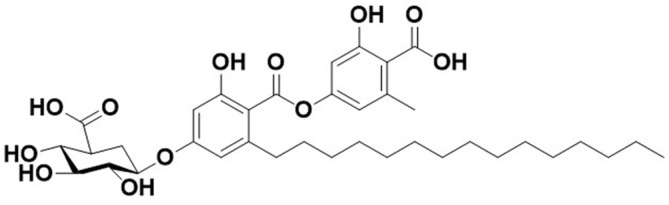
The structure of CRM646-A.

**Fig. 2:**
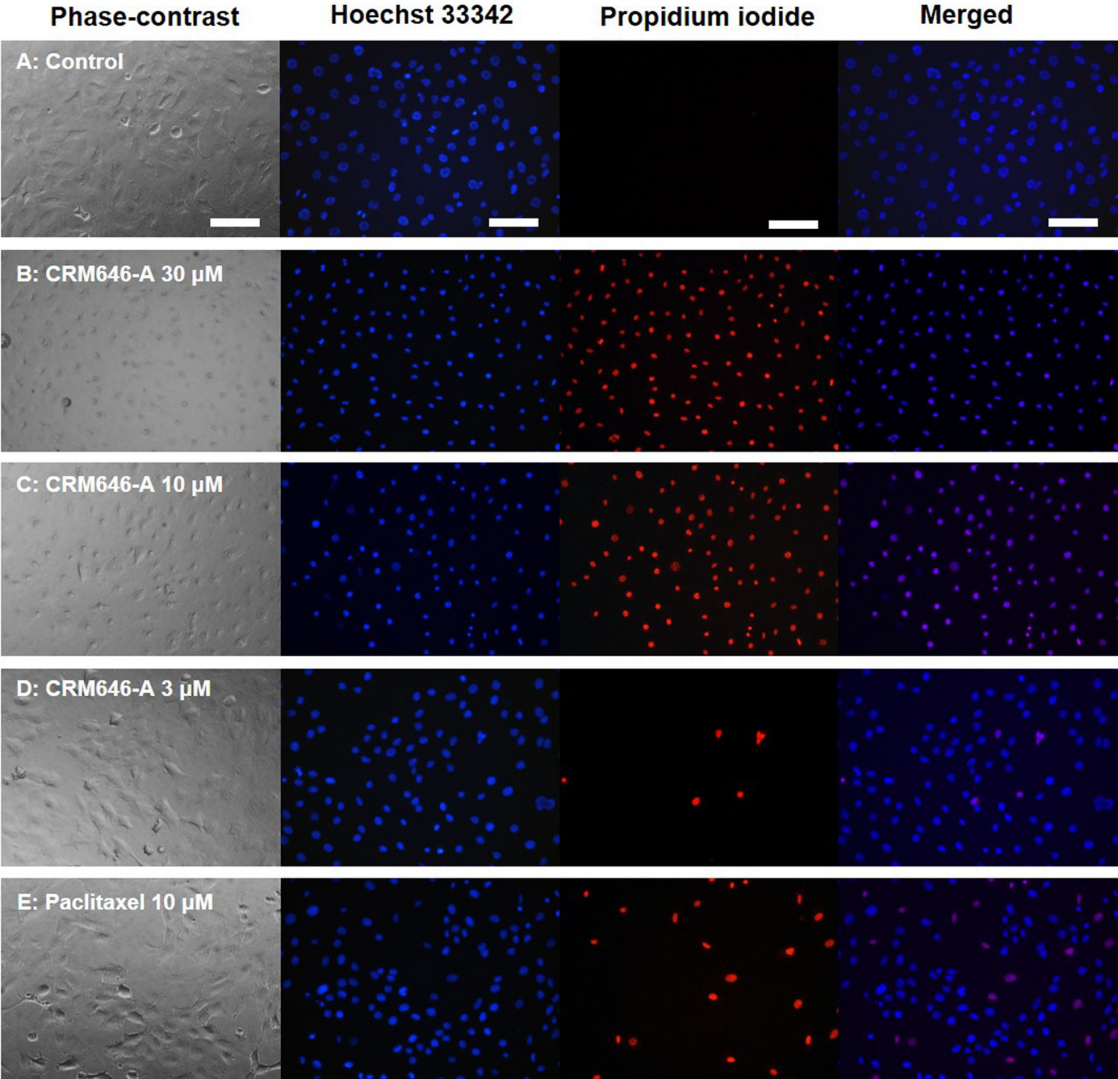
Test of dye exclusion for CRM646-A. 3Y1 cells were treated 15 min with CRM646-A or paclitaxel. These photographs showed cell membrane permeability and condensation of nuclei of 3Y1 cells after Hoechst 33343 (blue) and propidium iodide (red) staining in live cells for 5 min incubation. Each column shows (**A**), control; (**B-D**), 30, 10, or 3 μM of CRM646-A; (**E**), 10 μM of paclitaxel. White scale bars show 100 μm.

**Fig. 3:**
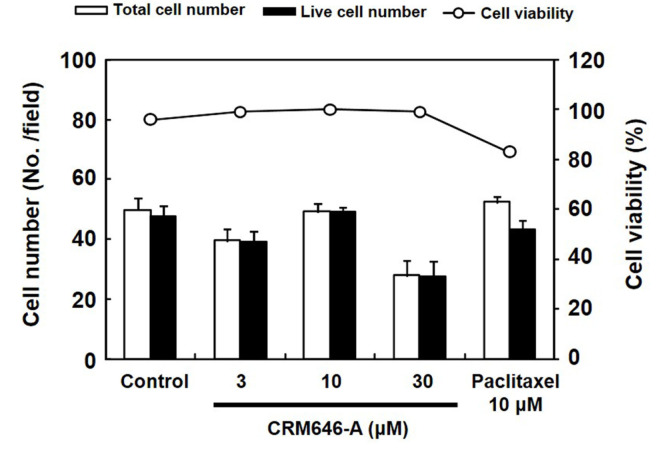
Cell viability experiment for PI-permeable cells. The effect of CRM646-A on cell viability was analyzed by using trypan blue exclusion method. 3Y1 cells were treated with 3, 10 or 30 μM CRM646-A and 10 μM of paclitaxel for 15 min. Values represent the means ± SD of quadruplicate samples. White bars show the total cell numbers. Bold bars show the live cell number. Open circles show the ratio of cell viability (%).

**Fig. 4:**
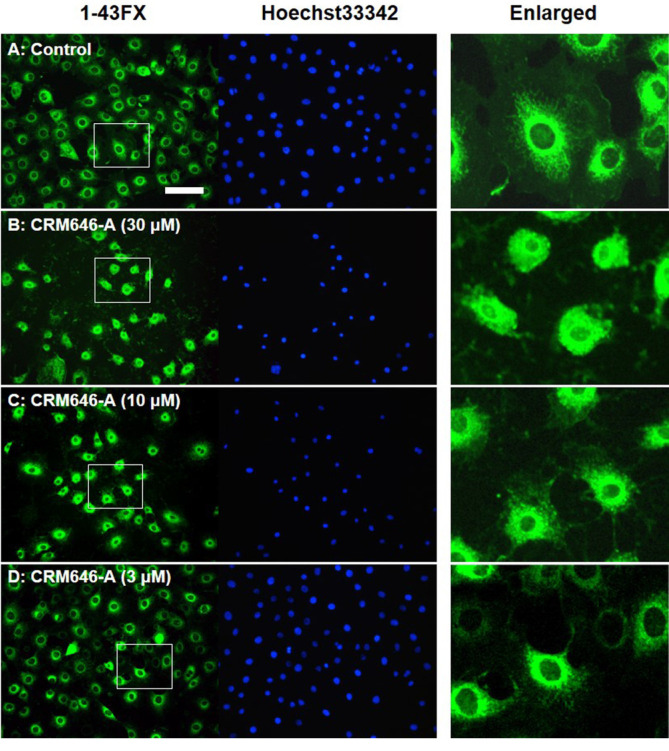
CRM646-A affects the morphology of cell membrane. These photographs show cell membrane and condensation of nuclei of 3Y1 cells after 1-43FX (green) and Hoechst 33343 (blue) staining in fixed cell by 3.7% formaldehyde. Each column shows (**A**), control; (**B-D**), 30, 10, or 3 μM of CRM646-A. White bar shows 100 μm scale. White square areas are enlargements of the photograph (4× magnification).

We previously identified some compounds and screened them using rat 3Y1 fibroblast cell morphological change assay to visualize phenotypes such as cell elongation, cellular outgrowth, and disruption of cell-to-cell interaction and to identify unique compounds [6]. In the course of this investigation, we observed that, after exposure to CRM646-A (Fig. 1) for 15 min, nuclei of rat 3Y1 fibroblast cells were clearly stained by propidium iodide and were mostly aggregated at a concentration of 30 and 10 µM (Figs. 2A-2D). At high concentration of paclitaxel, nuclei of rat 3Y1 fibroblast cells were moderately stained by propidium iodide and were partially aggregated (Fig. 2E). We examined whether CRM646-A induced cell death in a short time using trypan blue exclusion assay. CRM646-A did not induce cell death at 3, 10, or 30 µM but partially decreased the cell number at the concentration of 30 µM (Fig. 3). Hence, further investigation was conducted to elucidate the mechanism by which CRM646-A caused nucleus condensation in a short time. First, it was hypothesized that CRM646-A affected the morphology of the cell membrane. Therefore, an additional assay was performed to determine whether CRM646-A influenced the morphology of the cell membrane in rat 3Y1 fibroblast cells. As visualized by fluorescent 1-43FX dye, treatment with CRM646-A at both 30 and 10 µM concentrations induced disruption of morphology of the cell membrane with nucleus condensation after a 15-min treatment compared to the control (Fig. 4). Additionally, the structures of surrounding nuclei were dramatically disrupted in enlarged photographs (Fig. 4).

### Effects of Influx of Intracellular Ca^2+^ in Rat 3Y1 Fibroblast Cells on Treatment with CRM646-A

**Fig. 5:**
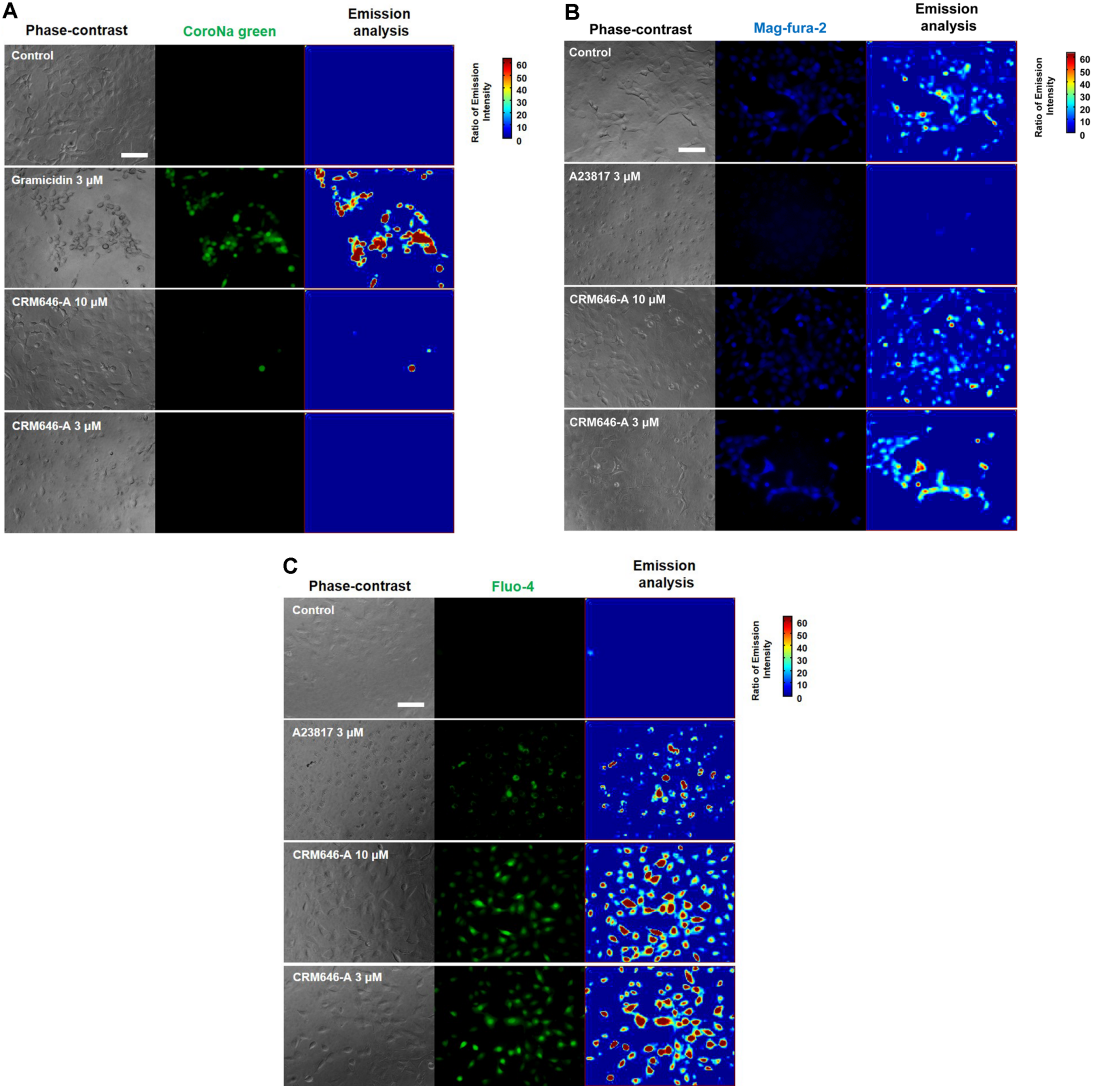
CRM646-A increases the intensity of Ca^2+^ in 3Y1 cells. After 3Y1 cells were treated for 30 min with CRM646-A or with gramicidin or A23187, we added fluorescence probes and incubated for 15 min. Left panels show phase contrast image, and middle panels show photographs of fluorescence images for Na^+^ (CoroNa green), Mg^2+^ (Mag-fura-2), or Ca^2+^ (Fluo-4) (**A**, **B**, and **C**, respectively). White bar shows 100 μm. Emission intensity was analyzed using MATLAB image in MATLAB R2011a (MathWorks, Natick, USA).

We monitored changes in environmentally sensitive fluorophores in living cells with three metal ions. The fluorescence emission intensities of Na^+^ and Mg^2+^ did not change during 30 min under treatment with CRM646-A at 10 µM concentration in living cells (Figs. 5A and 5B), but the fluorescence emission intensity of Ca^2+^ increased (Fig. 5C). However, CRM646-A did not show any F-actin aggregation, as of the last time CRM646-A-induced phenotype was regulated by Ca^2+^. This led us to hypothesize that Ca^2+^ concentration was correlated with CRM646-A-induced phenotypes such as inhibition of cell motility and growth, suppression of acinar morphogenesis, nucleus condensation, and disruption of plasma membrane.

### Effects of Suppression of ERK1/2 Phosphorylation and Nucleus Condensation with PD98059 on Treatment with CRM646-A

**Fig. 6:**
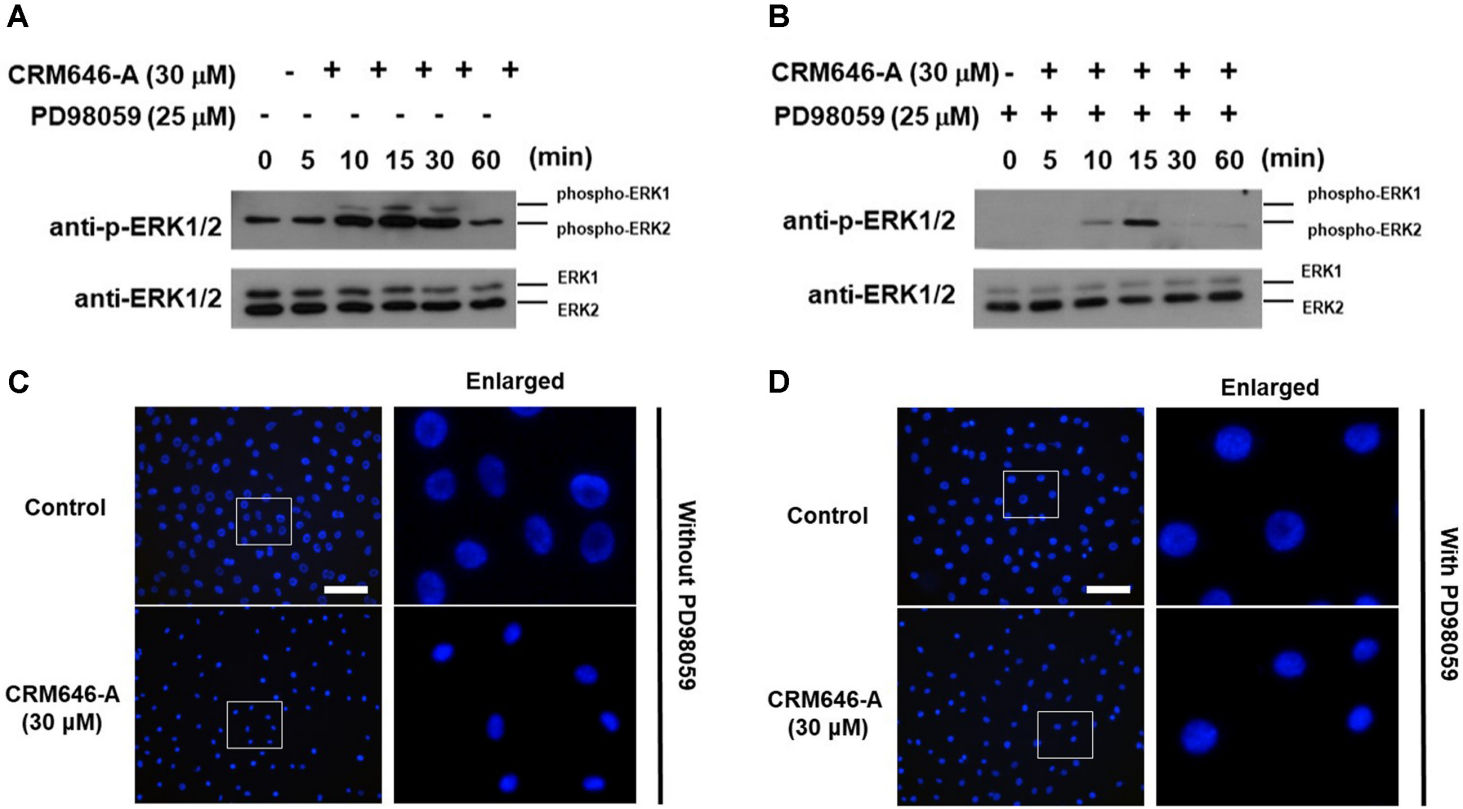
CRM646-A induces condensation of nuclei by ERK1/2 phosphorylation. (**A, B**) PD98059 inhibited the phosphorylation of ERK1/2 and nucleus condensation induced by CRM646-A. 3Y1 cells were pretreated for 60 min with 25 μM PD98059 before exposure to 30 μM of CRM646-A for 0, 5, 10, 15, 30 or 60 min. (**C, D**) After 3Y1 cells were treated 60 min with 25 μM of PD98059 before exposure to 30 μM of CRM646-A for 15 min, we added Hoechst 33343 (blue) staining in live cells for 5 min. Each column shows (**C**), without PD98059 and (**D**), with PD98059. White scale bars show 100 μm. White square areas are enlargements of the photograph (4× magnification).

Arachiche *et al*. demonstrated that ERK1/2 phosphory-lation was induced by Ca^2+^ ionophores in a specific manner [7]. Thus, we investigated whether CRM646-A induced ERK1/2 phosphorylation or not. We found that CRM646-A induced ERK1/2 phosphorylation from 10 to 30 min at 30 µM (Fig. 6A). In contrast, after pretreatment with 25 µM PD98059 for 60 min, ERK1/2 phosphorylation was clearly suppressed (Fig. 6B). We determined whether PD98059 inhibited CRM646-A-induced nucleus condensation in rat 3Y1 fibroblast cells. It was observed that 30 µM CRM646-A-induced nucleus condensation was partially suppressed by 25 µM PD98059 treatment (Figs. 6C and 6D). These results suggested that CRM646-A induced nucleus condensation in rat 3Y1 fibroblast cells in a ERK1/2 phosphorylation-dependent manner.

## Discussion

In this study, we investigated the cell basis of the inhibition of tumor metastasis in response to CRM646-A treatment. Approximately 20 years ago, Ahn *et al*. reported the antimetastatic effects of CRM646-A, but the causal relationship was not identified in detail. We have previously shown that CRM646-A exhibits heparinase inhibition and antimetastatic activity. Here, we found that nucleus condensation, disruption of plasma membrane, and increase in intracellular Ca^2+^ levels by CRM646-A were necessary for that biological activity in a short time compared with in vitro assay time of heparinase inhibition and antimetastatic test. We investigated whether CRM646-A correlated with morphological changes and nucleus aggregation by interaction with Ca^2+^ signaling without F-actin aggregation. These results suggested that CRM646-A showed different mechanisms as a Ca^2+^ inducer compared to violaceol-I and -II [6]. As shown in Fig. 2, we observed that CRM646-A-induced nuclei of rat 3Y1 fibroblast cells were clearly stained by propidium iodide, and nucleus condensation occurred within 15 min at 10 µM. Generally, live-dead staining is a widely used method to assess the viability of microbial and mammalian cells [8]. Propidium iodide does not normally permeate intact cell membranes, while the counterstain, Hoechst 33342, does. Both live cells and dead cells take up Hoechst 33342 and show blue fluorescence. When applied together with propidium iodide, dead cells will also be stained by propidium iodide and show red fluorescence. However, CRM646-A did not induce cell death at 3, 10, or 30 µM but partially decreased the cell number at the concentration of 30 µM by trypan blue assay (Fig. 3). From these observations, we hypothesized that CRM646-A might disrupt the plasma membrane of rat 3Y1 fibroblast cells. As shown in Fig. 4, when performing membrane staining by fluorescent 1-43FX dye, morphological changes of rat 3Y1 fibroblast cells were elicited by CRM646-A treatment without damaging actin and tubulin networks (data not shown). These data indicated that nuclei of rat 3Y1 fibroblast cells stained by propidium iodide in living cell plasma membranes might be impaired by CRM646-A treatment. However, the molecular mechanism of CRM646-A causing plasma membrane damage remains unclear. Recently, Signoretto *et al*. showed that a major mechanism accounting for cell shrinkage during eryptosis was the increase in Ca^2+^ levels [9]. As indicated in Fig. 5, we observed that CRM646-A increased the emission intensity of Ca^2+^ more than 30 times compared to the levels in live cells (Fig. 5C). CRM646-A did not increase or decrease the emission intensity of Na^+^ and Mg^2+^ compared to gramicidin and A23817 (Figs. 5A and 5B). Interestingly, CRM646-A preferentially increases Ca^2+^ levels by an unknown mechanism. Fei *et al*. also reported that Ca^2+^ fluxes regulated ERK kinase activation in mammalian cells [10]. As shown in Fig. 6A, CRM646-A transiently elicited ERK1/2 phosphorylation from 10 to 30 min in rat 3Y1 fibroblast cells. This phenotype synchronized nucleus condensation, disruption of plasma membrane, morphological changes, and Ca^2+^ uptake. Additionally, PD98059 strongly suppressed CRM646-A-induced phosphorylation of ERK1/2 by increasing Ca^2+^ levels in rat 3Y1 fibroblast cells (Fig. 6B). Interestingly, we observed that 30 µM CRM646-A-induced nucleus condensation was partially suppressed by 25 µM PD98059 treatment (Figs. 6C and 6D). Generally, the ERK pathway has been regarded as an anti-apoptotic kinase pathway [11, 12]. ERK functions as cytoprotective machinery against apoptosis triggered by many stresses [13, 14]. Likewise, tetracaine typically increases nucleus condensation, membrane blebbing, ERK1/2 activation, and Ca^2+^ concentration in PC12 cells [15]. These results and reported data indicated that inhibition of ERK1/2 activation by PD98059 increased CRM646-A-induced bioactivities, suggesting that ERK1/2 phosphorylation played a protective role in cellular survival pathway, and Ca^2+^ ionophore-like action might mediate CRM646-A bioactivities [9, 13, 15]. In summary, CRM646-A showed a unique biological activity potential that might be exploited to regulate Ca^2+^ signaling as a Ca^2+^ ionophore-like agent. CRM646-A can significantly induce nucleus condensation, disruption of plasma membrane, and morphological changes by increasing intracellular Ca^2+^ levels. These results suggest that CRM646-A-induced inhibition of cell migration, cell growth, and cell invasion in tumor cells correlates with nucleus condensation, disruption of plasma membrane, and morphological changes in response to the increase in Ca^2+^ levels. The biological effects of CRM646-A treatment may help provide further insight into the functions of Ca^2+^ signaling in tumor cells and lead to the development of new antimetastatic drugs.
